# Successful removal of a biliary metal stent using the stent-in-stent-in-stent technique

**DOI:** 10.1055/a-2113-7732

**Published:** 2023-09-15

**Authors:** Masato Matsuyama, Takeshi Okamoto, Tsuyoshi Takeda, Akiyoshi Kasuga, Takashi Sasaki, Masato Ozaka, Naoki Sasahira

**Affiliations:** Department of Hepato-Biliary-Pancreatic Medicine, Cancer Institute Hospital of Japanese Foundation for Cancer Research, Tokyo, Japan

A 60-year-old woman with unresectable pancreatic cancer underwent endoscopic biliary stenting with a covered self-expandable metal stent (SEMS) with an antireflux valve ( Fig. 1 ). She was readmitted 15 months later due to cholangitis. Cholangiography revealed contrast defect inside the stent, suspected to be the result of tumor invasion. As the stent could not be removed, the stent was trimmed below the ampulla. The removal attempt led to severe biliary hemorrhage that completely obstructed the endoscopic view, and therefore a covered SEMS was deployed under fluoroscopic guidance, leading to successful hemostasis and biliary drainage ( Fig. 2 ). The two stents could not be removed when cholangitis recurred just 1 month later, possibly owing to the short duration of the stent-in-stent configuration. Another covered SEMS with an antireflux valve was deployed inside the two existing stents, as the patient clearly had longer time to recurrent biliary obstruction when an antireflux value was present ( Fig. 3 ).

**Fig. 1:**
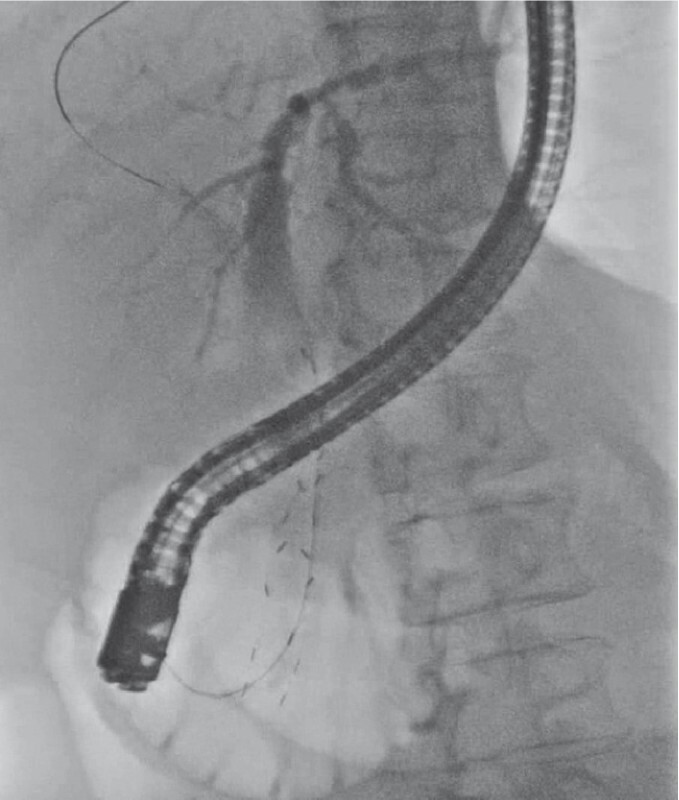
Initial placement of a duckbill-type self-expandable metal stent (Duckbill IT Biliary Stent: Kawasumi Laboratories Inc., Tokyo, Japan).

**Fig. 2:**
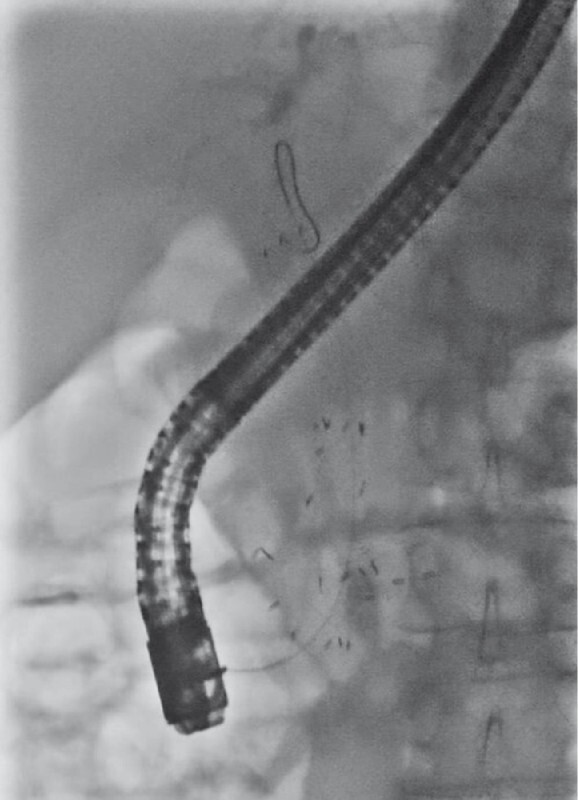
Stent-in-stent placement of a fully covered self-expandable metal stent (HANAROSTENT; Boston Scientific Corp., Marlborough, Massachusetts, USA).

**Fig. 3:**
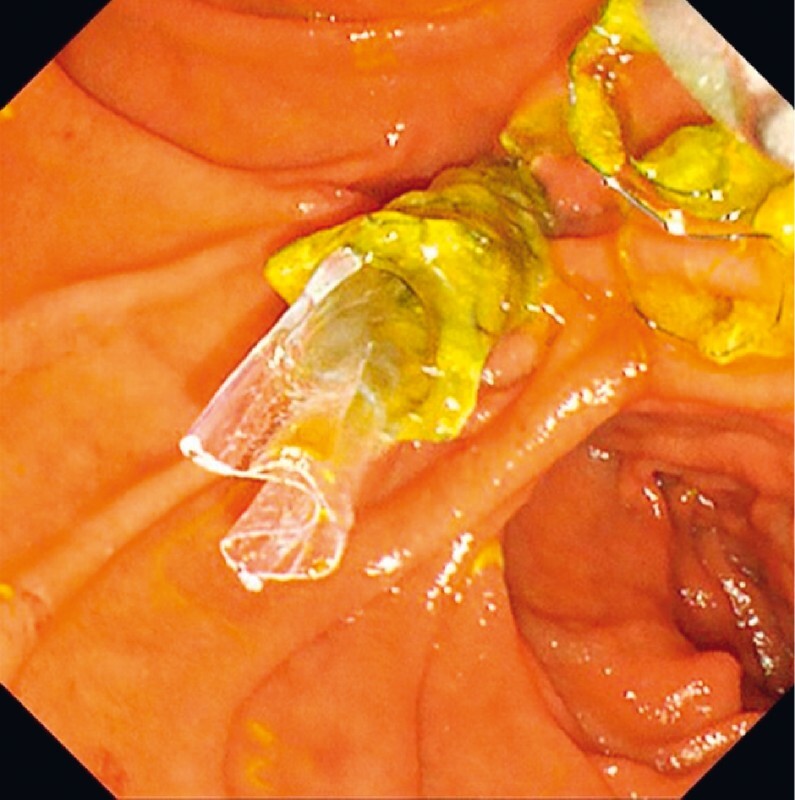
Stent-in-stent-in-stent placement of a duckbill-type self-expandable metal stent (Duckbill Biliary Stent; Kawasumi). The antireflux value can be seen protruding from the second stent.

The patient presented with a third episode of cholangitis 10 months later. Endoscopic retrograde cholangiopancreatography revealed stent obstruction due to biliary stones and debris. When sweeping the lumen of the three stents using stone extraction balloons, all three stents gradually migrated toward the duodenum. We therefore removed all three stents together using an endoscopic snare ( Fig. 4 ), which was successfully performed with no resistance ( Fig. 5 ). A new laser-cut covered SEMS with an antireflux valve was placed, leading to resolution of the patient’s symptoms ( Video 1 ).

**Fig. 4:**
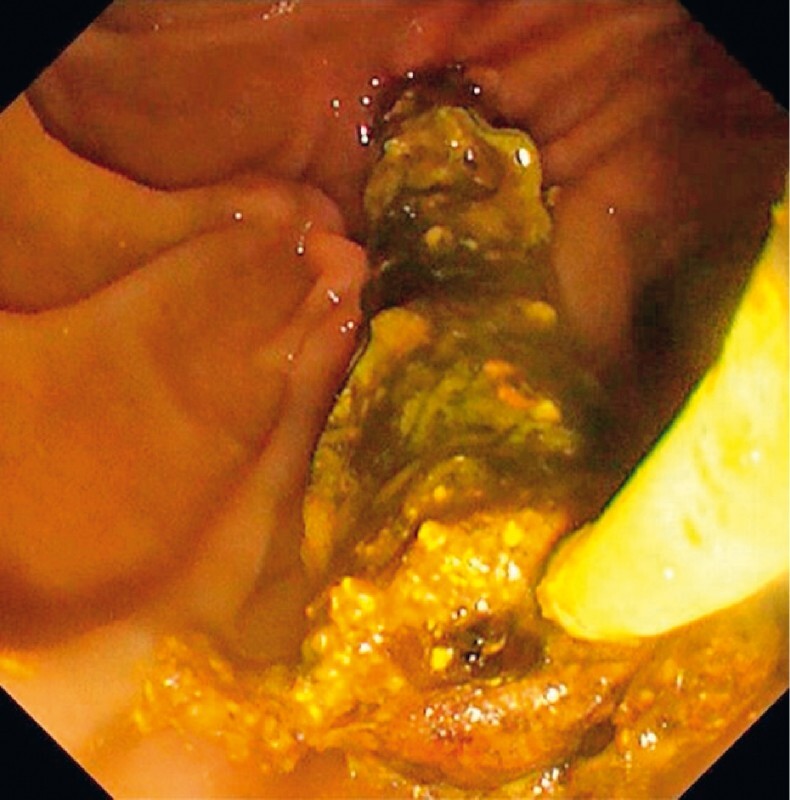
Sweeping with a stone extraction balloon (Multi-3V Plus, 15 mm; Olympus Medical Systems Corp., Tokyo, Japan) led to migration of the three stents toward the duodenum.

**Fig. 5:**
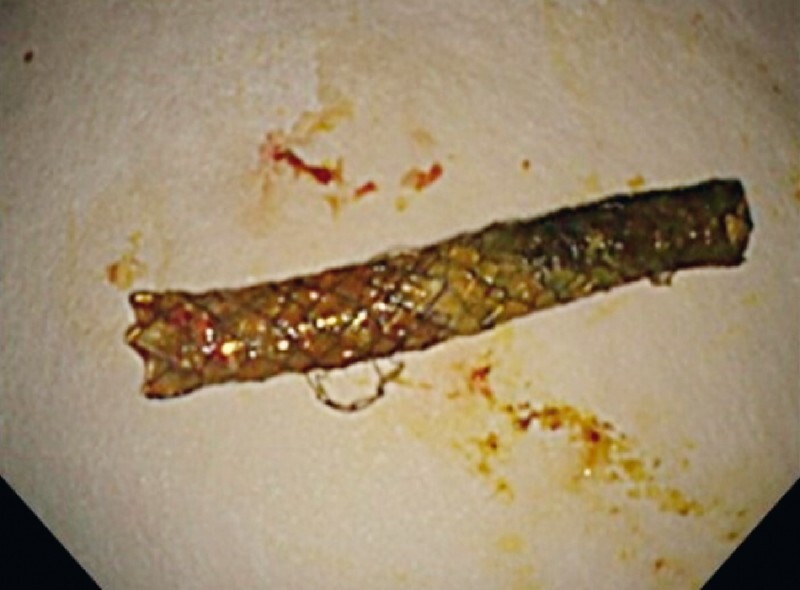
The stent-in-stent-in-stent complex was removed with a snare.

**Video 1** Successful removal of a biliary metal stent using the stent-in-stent-in-stent technique.


Successful removal of both uncovered 1 2 and covered SEMSs 3 has been reported using the stent-in-stent technique. The duckbill-type stent is a novel covered SEMS with an antireflux valve 4 . The removal of this kind of SEMS can be difficult 4 5 . When the stent-in-stent technique proves unsuccessful, the addition of a third stent may facilitate removal.

Endoscopy_UCTN_Code_CPL_1AK_2AD

## Notes

Endoscopy E-Videos

https://eref.thieme.de/e-videos
*E-Videos* is an open access online section of the journal *Endoscopy* , reporting on interesting cases and new techniques in gastroenterological endoscopy. All papers include a high-quality video and are published with a Creative Commons CC-BY license. Endoscopy E-Videos qualify for HINARI discounts and waivers and eligibility is automatically checked during the submission process. We grant 100% waivers to articles whose corresponding authors are based in Group A countries and 50% waivers to those who are based in Group B countries as classified by Research4Life (see: https://www.research4life.org/access/eligibility/ ). This section has its own submission website at https://mc.manuscriptcentral.com/e-videos
